# Global Radiative Impacts of Black Carbon Acting as Ice Nucleating Particles

**DOI:** 10.1029/2020GL089056

**Published:** 2020-10-12

**Authors:** Zachary McGraw, Trude Storelvmo, Bjørn Hallvard Samset, Camilla Weum Stjern

**Affiliations:** ^1^ Department of Geosciences University of Oslo Oslo Norway; ^2^ Center for International Climate and Environmental Research‐Oslo (CICERO) Oslo Norway

**Keywords:** black carbon, climate, aerosol indirect effects, cirrus, climate modeling

## Abstract

Black carbon (BC) aerosols from incomplete combustion generally warm the climate, but the magnitudes of their various interactions with climate are still uncertain. A key knowledge gap is their role as ice nucleating particles (INPs), enabling ice formation in clouds. Here we assess the global radiative impacts of BC acting as INPs, using simulations with the Community Earth System Model 2 climate model updated to include new laboratory‐based ice nucleation parameterizations. Overall, we find a moderate cooling through changes to stratiform cirrus clouds, counteracting the well‐known net warming from BC's direct scattering and absorption of radiation. Our best estimates indicate that BC INPs generally thin cirrus by indirectly inhibiting the freezing of solution aerosol, with a global net radiative impact of −0.13 ± 0.07 W/m^2^. Sensitivity tests of BC amounts and ice nucleating efficiencies, and uncertainties in the environment where ice crystals form, show a potential range of impacts from −0.30 to +0.02 W/m^2^.

## Key Points

- Anthropogenic black carbon (BC) thins cirrus clouds by inhibiting homogeneous nucleation, inducing moderate global cooling
- The cooling impact saturates at high BC number and nucleating efficiency, due to cirrus thickening via enhanced heterogeneous ice formation
- BC cooling by cirrus thinning requires colocation of the aerosol with abundant, medium‐thickness homogeneously formed cirrus

## 1 Introduction

Black carbon (BC) influences Earth's climate through a variety of mechanisms (Bond et al., 2013; Peng et al., 2016), many of which are still poorly constrained. BC has a “very high probability” of warming Earth's climate overall, primarily through its ability to absorb solar radiation (Bond et al., 2013). Recent multimodel studies have consistently quantified the surface warming from early 21st century anthropogenic BC emissions to be around +0.1°C (Baker et al., 2015; Samset et al., 2016; Stjern et al., 2017). However, there are still considerable model differences, linked partly to the low confidence in assessments of BC's diverse roles in clouds (Bond et al., 2013; Stjern et al., 2017). One such mechanism is BC's ability to act as surfaces where ice crystals form at relatively low saturations (ice nucleating particles [INPs]). Ice clouds have an important role in Earth's radiative balance by both reflecting incoming shortwave (SW) radiation back to space and absorbing outgoing longwave (LW) radiation and have overall been found to warm the atmosphere by as much as 5.1 ± 3.8 W/m^2^ globally (Hong et al., 2016). Depending on circumstances, the presence of INPs can either increase or decrease the number density of ice crystals in clouds. Here the impact of BC INPs depends on the number of BC particles at ice cloud altitudes, their ice nucleating efficiency, and their influence on processes that compete for water vapor. Larger (lower) densities of ice crystals typically cause optically thicker (thinner) clouds. INP changes may either strengthen or weaken ice cloud warming effects depending on the balance between LW and SW impacts.

**Figure 1:**
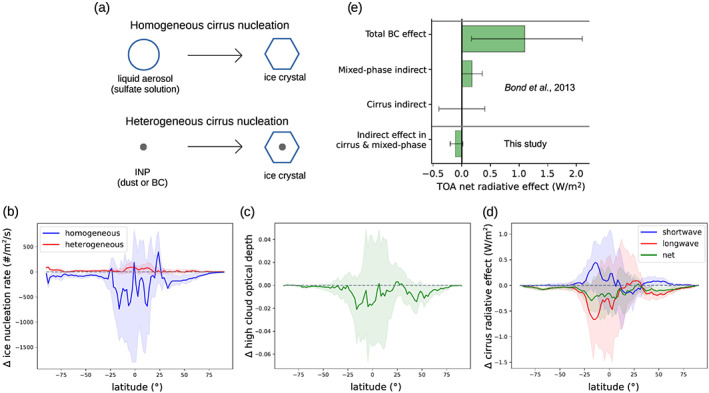
Impacts of anthropogenic black carbon INPs on cirrus clouds and radiation. Shown in counterclockwise order are a schematic of the two competing ice nucleation mechanisms in cirrus (a), simulated anthropogenic BC impacts on heterogeneous and homogeneous cirrus nucleation rates (vertically integrated, in number of events per m^2^ per second) (b), impacts on visible shortwave optical depth integrated over high clouds (<400 mb) (c), impacts on top‐of‐atmosphere cirrus radiative effects (in W/m^2^) (d), and a comparison between our finding of the total net BC INP effect (dominated by cirrus effects) compared with values from Bond et al. (2013) (e). Shading in (b)–(d) shows the 25th and 75th percentiles among columns within each zonal band. Model averages of these variables are shown in Figure S1. Bounds on our result in (e) are the range of results among sensitivity simulations, while all effects shown are for anthropogenic BC.

While, in some cases, the presence of INPs will optically thicken ice clouds by enabling crystals to form on their surfaces (*heterogeneous* nucleation), among cirrus that initially form through the freezing of liquid aerosol at high saturations (*homogeneous* freezing/nucleation), the impact of INPs tends to be the opposite (Sullivan et al., 2016; Zhao et al., 2018). Figure 1, 2 shows a schematic of the two mechanisms. INP presence enables water vapor to be consumed as new ice crystals form heterogeneously and grow. By acting as a sink of water vapor, these INPs can prevent conditions from reaching the saturations necessary for homogeneous freezing. Due to the greater atmospheric abundance of solution droplets than INPs, INP increases often result in cirrus optical thinning by decreasing the number and increasing the size of ice crystals. Whether INPs cause thinning or thickening of cirrus depends on which of the nucleation mechanisms dominates, which itself depends on cirrus updraft speeds, the presence of INPs both new and prior (Penner et al., 2018), availability of solution droplets for homogeneous freezing, and the type of cirrus affected (since radiative effects vary for instance by cloud top temperature and underlying albedo; Corti & Peter, 2009). Global assessments of INP impacts are limited by inadequacies in global climate models, which typically have insufficient resolutions to resolve the vertical motions that both induce high saturations and bring aerosol to ice cloud altitudes, relying instead on parameterizations. These issues are compounded with difficulties in simulating aerosol sinks, disagreement between laboratory findings of ice nucleating efficiency (Ullrich et al., 2017), and limited aerosol complexity in models (Liu et al., 2016).

BC number concentrations at high altitudes are difficult to assess due to large uncertainties in emissions, transport, and lifetime (Bond et al., 2013; Boucher et al., 2013, 2016; Lund et al., 2018). Laboratory experiments have shown large variation in BC ice nucleating efficiency by particle morphology, size, and aging but have evidenced their potential especially in cirrus clouds (Kanji et al., 2017; Mahrt et al., 2018, 2020; Ullrich et al., 2017). Past model‐based estimates of anthropogenic BC INP radiative impacts have ranged from as much as −0.4 to +0.4 W/m^2^ in cirrus to +0.18 ± 0.18 W/m^2^ in mixed‐phase clouds (Bond et al., 2013), with a more recent assessments indicating near −0.3 W/m^2^ in cirrus (Penner et al., 2018).

This study assesses the radiative impacts of anthropogenic BC INPs using the atmosphere component of the Community Earth System Model (CESM) v2.0.1 global climate model modified to include laboratory‐based parameterizations of BC ice nucleating potential (Ullrich et al., 2017). Simulations were designed to evaluate effective radiative forcings (ERFs), a standard forcing measure that is a useful predictor of global mean near‐surface air temperature change (Myhre et al., 2013). We also conducted sensitivity tests to account for how many of the abovementioned uncertainties may influence BC INP impacts. The result is a comprehensive bounding of the potential climate implications of anthropogenic BC emissions through INP interactions, taking into account the best available knowledge on aerosol‐cloud microphysics and Earth System modeling.

## 2 Methods

Results are based on output from simulations running the CESM2.0.1 (Danabasoglu et al., 2020) model at 1.9° × 2.5° resolution with 32 vertical levels, using the atmosphere component CAM6 and fixed sea surface temperatures. Simulations used the MAM4 modal aerosol module (Liu et al., 2016), which includes two BC aerosol species, with BC emitted in primary mode and transferred to the accumulation mode as it is aged with coatings of sulfate aerosol. BC emissions were fed in from files containing preindustrial (PI; 1750) and present‐day (PD; 2000) mass and number surface fluxes (Hoesly et al., 2018). All simulations included BC emissions from fossil fuel and biofuel sources, as well as changes in biomass burning BC emissions since PI times. In a sensitivity test, we also added aircraft soot, which was not included in either the core simulations or other sensitivity tests.

Ice nucleation processes on BC and mineral dust INPs were replaced with the parameterizations of Ullrich et al. (2017). These parameterizations were derived from laboratory experiments over 11 years that used the Aerosol Interaction and Dynamics chamber at cirrus and mixed‐phase temperatures. In Ullrich et al. (2017), heterogeneously formed ice crystal numbers are calculated as functions of temperature, ice saturation ratio, and aerosol surface area density, using the concept of ice nucleation active surface site density (Hoose & Möhler, 2012). Since BC INPs are known to become less active as the aerosol become coated with solution droplets, we scaled down INP efficiency of accumulation mode BC using a 1% ice nucleation active surface site scaling as in Ullrich et al. (2019). To enable heterogeneous nucleation from Ullrich et al. (2017) to interactively compete with homogeneous freezing in cirrus, we embedded the Ullrich et al. (2017) parameterizations in the Barahona and Nenes (2009) cirrus nucleation scheme. This scheme approximates the analytical solution of cloud parcel equations considering both homogeneous and heterogeneous nucleation and was ideal for use here because it allows the heterogeneous nucleation function to be customized.

For comprehensiveness, we implemented the Ullrich et al. (2017) parameterizations in both cirrus and mixed‐phase clouds. We further implemented these parameterizations in both stratiform clouds and deep convective cores (using Morrison & Gettelman, 2008, and Song et al., 2012, microphysics, respectively). However, BC INPs in stratiform cirrus were found to dominate the total net radiative effects (see supporting information Table S1), so we primarily focus on impacts in these clouds. BC is now understood to be an ineffective INP at mixed‐phase cloud temperatures (Mahrt et al., 2018; Vergara‐Temprado et al., 2018). In convective cores, INPs may have opposite effects for moderate and strong convective systems (Zhao et al., 2019), while local nucleation at cirrus levels tends to be inhibited by an abundance of homogeneously frozen droplets lifted from below.

For each model configuration, we used four simulations to determine the impacts of anthropogenic BC INPs. A first pair of runs simulated PI and PD levels of BC with the abovedescribed setup, while a matching pair was run with all ice nucleation on BC turned off. We then calculated the ERF due to anthropogenic BC INPs while excluding other mechanisms (i.e., direct, semidirect, and warm cloud effects). This was done by comparing net cloud radiative effects between simulations as follows:

(1) $$\begin{array}{c} {\mathrm{ERF}}_{\text{anthroBCINPs}}=\left({\mathrm{CRE}}_{\text{PDBC}}-{\mathrm{CRE}}_{\text{PIBC}}\right)-\left({\mathrm{CRE}}_{\text{PDBC}}^{\text{NoBCINPs}}-{\mathrm{CRE}}_{\text{PIBC}}^{\text{NoBCINPs}}\right) \end{array}.$$

**Figure 2:**
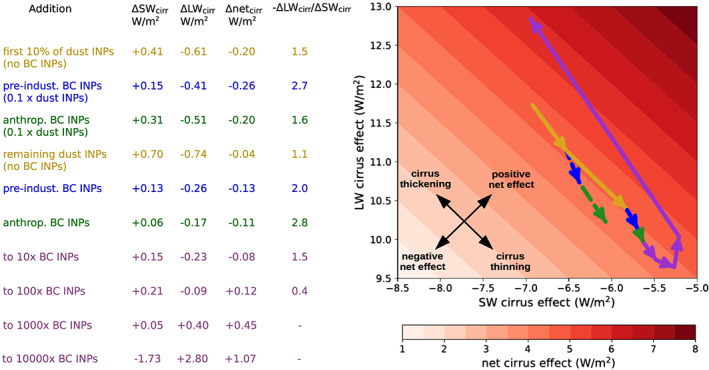
Modeled changes to global cirrus radiative effects as INPs are added. Marked are the cirrus radiative effects before and after each INP addition, as arrow tails and heads, respectively. First, dust INPs are added with no other INPs present (dark yellow lines, initially 10% of the default number of dust INPs followed by the remaining 90%), then BC natural (green) and anthropogenic (blue) INPs are added, and lastly, all BC INP number concentrations are progressively increased to 10× the previous value (purple). Initial BC increases are represented twice, for simulations having the default amount of dust INPs (solid lines) and 10% as many (dashed). Background color shows net cirrus effects, being the sum of SW and LW effects. Black arrows serve as a guide to (i) the directions of positive and negative net forcings and (ii) the directions of cirrus radiative thickening or thinning equal in the shortwave and longwave (zero net forcing).

Equivalent calculations were completed for SW and LW BC INP forcings, and all forcings were additionally calculated for cloud radiative effect changes in cirrus only. The cirrus forcings were calculated to better understand the response in cirrus and mirrored standard cloud radiative diagnostics while only considering clouds in grid cells colder than −38°C (based on the method in Gasparini & Lohmann, 2016). In addition to the core and sensitivity simulations, four additional simulations were ran to develop an understanding of diverse INP changes (Figure 2), and four more were used to separate BC effects by cloud type (Table S2). All simulation sets and stand‐alone runs are briefly described in Table S1, and additional simulation details are presented in Text S1.

## 3 Results

### 3.1 Core Estimate of Anthropogenic BC INP Effect

Our core result is an estimate of the current ERF due to BC particles acting as INPs. Comparing simulations (see section 2) showed anthropogenic BC INPs to cause a net radiative forcing of −0.13 ± 0.07 W/m^2^ globally and is expected to represent a cooling effect at the surface and lower troposphere. The presented uncertainty is the standard deviation among annual average forcings for 5 simulated years. This result did not factor in aircraft soot emissions, which we found to weaken the forcing to a small extent within the uncertainty range. In the next section we attribute the core result to a reduction in the simulated optical thickness of cirrus clouds, which generally warm the climate. Our result is shown in Figure 1e, in comparison with prior research, along with an uncertainty estimate from the range of sensitivity tests detailed below. Figure 1d shows BC INP impacts specifically on cirrus radiative effects, which we found to dominate the total radiative response. Impacts of BC INPs in mixed‐phase clouds and deep convective cores were not found to be significant (see Table S2), and hence, we focus on stratiform cirrus for the remainder of this letter. We first describe how and why a moderate cooling impact occurs and then discuss to what extent this outcome is robust.

### 3.2 Process Understanding

The CESM2 simulations used here represent the chain of interactions from INP presence to associated radiative impacts. Depending on whether INPs optically thicken or thin clouds and whether this primarily impacts SW or LW radiation, INPs can cause either a warming or cooling effect. In our core simulations, anthropogenic BC impacts on net cirrus radiative effects (Figure 1d) roughly match the zonal structure of changes in homogeneous nucleation rates (Figure 1b) and high cloud optical depth (Figure 1c), suggesting a connection. Averaged globally, anthropogenic BC induces a decrease in cirrus nucleation rates and an optical thinning of cirrus. Impacts vary considerably by location, with both moderate cirrus thickening and thinning occurring (as seen in the percentile bounds in Figure 1c). Across latitudes, averaged anthropogenic BC INP effects on high cloud optical depth roughly correspond to changes in homogeneous (not heterogeneous) nucleation rates, showing the dominant influence of homogeneous nucleation with this model setup. In the southern tropics, radiative cooling is further enhanced by a BC INP‐induced reduction in high cloud cover (Figure S2e).

BC INP impacts on cirrus radiative effects are the strongest, but also the most varied, in the tropics. SW effects are strong in this region partly because the underlying surface tends to be dark (Figure S2b), while LW effects are strong in part due to the cold cloud temperatures (and thus lower reemission of LW radiation to space) (Figure S2c). Local variations in these properties enable a change in cirrus optical depth to have highly varied effects depending on the cirrus affected. The tropics show the largest declines in total cirrus nucleation rates due to anthropogenic BC (Figure 1b). This is unsurprising since homogeneous nucleation is strongly prevalent in this region (Figure S1a) of frequent cirrus (Figure S2a), where convective events lift water, INPs, and liquid aerosol to high altitudes. These conditions provide an environment where modest shifts in the competition between homogeneous and heterogeneous nucleation can have large impacts. In a minority of high tropical cirrus, BC INPs counterintuitively cause homogeneous nucleation rates to increase. This is likely due to the BC INPs having secondary effects by influencing cirrus temperatures (Figure S2g) and updraft speeds (Figure S2h). The southern extratropics are relatively isolated from anthropogenic BC sources, and hence, cirrus thinning is relatively weak here. However, for these cirrus clouds, LW radiative effects tend to dominate—due in part to high underlying albedo (Figure S2b)—so their reduction does significantly alter the local radiative balance. In the northern extratropics, anthropogenic BC thins cirrus at nearly all locations despite the abundance of cirrus formed by heterogeneous nucleation, as the impact on homogeneous nucleation remains stronger. Hence, noticeable net cooling occurs at nearly all latitudes.

While our simulations show anthropogenic BC INPs to overall thin cirrus and cause cooling, the outcome depends greatly on the number and efficiency of these INPs as well as those of other colocated INPs, such as dust. Figure 2 shows the changes that occur to global‐mean cirrus radiative effects as various INP additions are simulated. The addition of anthropogenic BC—shown in simulations both with default and low dust INP concentrations—causes a negative forcing by weakening cirrus LW effects more than SW effects, as is seen by comparing the slope of the arrows with the guidelines (and as −ΔLW_cirr_/SW_cirr_ > 1 in the accompanying table). However, as further BC INPs are added, the dominance of the LW impact diminishes (lowering −ΔLW_cirr_/SW_cirr_). This weakens the net impact of each addition and eventually results in positive forcings when the SW weakening comes to dominate (−ΔLW_cirr_/SW_cirr_ < 1). Similarly, impacts are weakened by the presence of other colocated INP species, as shown by anthropogenic BC INPs causing nearly twice the forcing when dust INP number is reduced to 10% globally. Anthropogenic BC INPs induce a significant negative forcing because their emission sources allow them to reach some cirrus otherwise lacking sufficient INPs to saturate (or reverse) the effect. Mineral dust, the most recognized other INP, is primarily emitted from desert regions far from major anthropogenic sources and hence likely does not reach the same cirrus as anthropogenic BC. Additionally, BC tends to be smaller than dust and hence capable of being transported further without falling out. Once an initial amount of dust INPs are globally present, adding BC INPs has a substantially more LW dominant and net negative effect than adding further dust INPs.

Differences in affected homogeneously and heterogeneously formed cirrus may explain why the initially negative INP forcing diminishes and reverses as more INPs are added. Initial INPs have a relatively large impact on homogeneously formed cirrus radiative effects by preventing many freezing events per INP, inducing a negative forcing while the thickening effect in heterogeneously formed cirrus is too weak to be comparable. Gradually, as large‐scale INP additions are made, homogeneously formed cirrus become less abundant, and their responses are outpaced by the net positive effect from thickening heterogeneously formed cirrus. Hastening this shift, changes to the thickness of heterogeneously formed cirrus tend to induce more LW‐dominant effects than those to the relatively thick homogeneously formed cirrus, since cloud LW radiative effects saturate in thick cirrus over a range of optical depths where SW effects do not (Choi & Ho, 2006; Hong et al., 2016). Ultimately, thickening of heterogeneously formed cirrus results in a positive net effect even, while thinning of homogeneously formed cirrus may continue to dominate the globally averaged SW and LW responses (possibly explaining why in Figure 2 INP additions induce counterclockwise rather than linear movements). We thus find that anthropogenic BC INPs induce a cooling effect because they encounter and thin cirrus formed through homogeneous nucleation.

### 3.3 Sensitivity Tests

**Figure 3:**
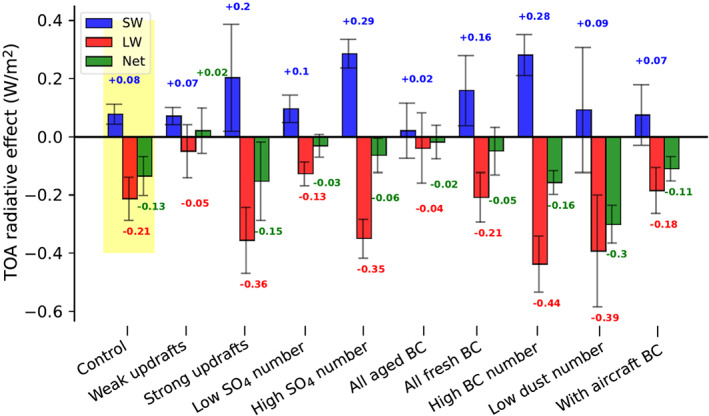
Global effective radiative forcings from anthropogenic BC INPs. Shown are outcomes from the control simulation set and all sensitivity tests. Uncertainties indicate one standard deviation among annual averages throughout the 5 year simulation period.

As model uncertainties can influence the balance between the warming and cooling effects of anthropogenic BC INPs, we ran sensitivity tests to determine the extent to which impacts could plausibly differ from our core result. ERFs from all sensitivity tests are shown in Figure 3. These tests represent a diverse range of plausible INP and cirrus characteristics. For one, BC INP number and efficiency are poorly constrained in simulated cirrus. Assuming a high bound on anthropogenic BC INP number concentrations of nearly 10× those in the default model (resembling the spread of middle‐upper tropospheric BC mass among models; Samset et al., 2013; Schwarz et al., 2017) increased the global forcing by 16%, indicating a plausibly larger cooling effect but also that the impact of each INP added diminishes as more are added. Adding aircraft soot emissions (not otherwise simulated) affects cirrus primarily at Northern Hemisphere latitudes where heterogeneous nucleation already dominates, causing this INP source to primarily enhance cirrus SW and LW effects (Figure S4). While aircraft soot thus counterbalances the impacts of BC INPs on homogeneously formed cirrus with a slight positive net radiative impact, this source is not abundant enough to significantly weaken the anthropogenic BC INP forcing. Simulating BC INPs as if they all age into relatively inefficient INPs when emitted (1% as efficient as fresh) unsurprisingly rendered the forcing insignificant. More surprisingly, treating all BC as fresh INPs similarly reduced the net cooling effect by inducing SW thinning with little LW effect. For maximal cooling, INPs would cause cirrus to thin without substantially forming or thickening heterogeneously formed cirrus. Hence, regardless of possibilities for more numerous or effective BC INPs (largely equivalent) than assumed in the core finding, the narrow range of suitable INP concentrations for additional BC INPs to induce cooling (see Figure 2) considerably constrains the potential for an enhanced cooling. Similarly, a strong warming effect is difficult to achieve since it requires very high concentrations or efficiencies of BC INPs.

Anthropogenic BC INP impacts could hypothetically be stronger if the affected cirrus were different from those in our control simulation in ways that further enable BC INPs to thin cirrus more dominantly in the LW than SW. We tested this possibility by altering uncertain parameters that influence the balance between heterogeneous and homogeneous nucleation events. This includes updraft variability, which is crudely parameterized in the model, and the availability of both sulfate aerosol for homogeneous nucleation and additional INP species. Rerunning the model with an increased cirrus updraft spread enables a greater number of cirrus to reach the saturations necessary for homogeneous sulfate nucleation, increasing the frequency and extent of these events. In contrast, enhancing sulfate availability enabled more homogeneous nucleation to occur only in the few cirrus where homogeneous nucleation is already strong enough to exhaust the supply of sulfate without the addition. These adjustments had opposite effects, with increased updraft variability resulting in a stronger negative forcing while increased sulfate availability reduced the forcing (+13% and −52% net forcings compared to the control, respectively). The difference is presumably because the sulfate increase thickens cirrus that are already LW saturated, while the updraft change enables the INPs to thin less thick cirrus. As seen in Table S3, the model setup with the updraft change has the strongest cirrus net radiative effect (+5.12 W/m^2^, compared to +4.34 W/m^2^ in the control) among sensitivity tests, while that with the sulfate change has the weakest (+3.83 W/m^2^). This follows the expectation that reducing the most warming cirrus would have the most cooling effect while thinning the least warming would cause less cooling. Similar sensitivity runs with the opposite changes (less updraft variability and less available sulfate) unsurprisingly led to weak INP radiative impacts. An additional test incorporated less numerous (10%) dust INPs in simulations. This roughly doubled the net anthropogenic BC forcing, being the highest enhancement among sensitivity tests and demonstrating the abovementioned concept of the strongest negative net effects when few INPs are present.

## 4 Conclusions

Our simulations showed PD anthropogenic emissions of BC aerosols to generally thin cirrus clouds through their role as INPs, resulting in a moderate net negative radiative forcing at the top of the atmosphere. Such a forcing is expected to cool Earth's surface and lower‐middle troposphere, though—as seen in other studies of high altitude BC forcings (Samset & Myhre, 2015; Stjern et al., 2017)—the temperature response may not readily scale with the forcing. This study was unique in its consideration of BC INPs acting in diverse cloud types, which included stratiform and convective clouds at both mixed‐phase and cirrus temperatures. Convective clouds in particular have been ignored in previous studies. The use of contemporary laboratory fittings for BC ice nucleating ability showed BC to be a weak INP at mixed‐phase temperatures with negligible net radiative impacts, a result that disagrees with the consensus of a warming BC INP effect in these clouds (Bond et al., 2013) (see Figure 1e). Overall, anthropogenic BC INPs caused a negative forcing that was primarily due to their presence in stratiform cirrus within environments that in their absence were capable of supporting homogeneous freezing. Our result of a small cooling in stratiform cirrus resembled findings in some studies (Gettelman et al., 2012; Penner et al., 2018) though in bounding this estimate we diverged from the consensus that a warming effect is as likely (Bond et al., 2013) (Figure 1e).

Among tests representing a range of plausible heterogeneous and homogeneous ice nucleation strengths and sensitivities to BC INPs, anthropogenic BC INPs were found to have an ERF ranging from −0.30 to +0.02 W/m^2^. The methods used to establish this range did have some limitations that we note here. While we tested BC INP effects for a comprehensive range of realizations, plausible outcomes may not have been entirely constrained due to the reliance of sensitivity tests on one model and uncertain parameter bounds. All simulations had cirrus radiative effects (Table S3) well within the global mean observational bounds of Hong et al. (2016), indicating potentially more uncertainty in cirrus properties than tested. As with other studies that simulated BC INP effects without observations to directly corroborate findings, confidence in the stated bounds remains low. We did not incorporate the possibility that BC aged through cloud processing may substantially enhance its INP efficiency (Mahrt et al., 2020), which could either strengthen or reduce the forcing. Conversely, as many types of BC have been found in laboratory experiments to be ineffective INPs (Mahrt et al., 2018) and BC at cirrus levels may not match the samples used to create parameterizations, there is a possibility that BC is instead a weaker INP than represented here. Lastly, while simulations tested BC INP effects in a comprehensive range of ice clouds, they relied on a single model treatment of ice optical properties and some clouds were likely not well represented due to the coarse model resolution.

We expect that the stated range represents a realistic bound on the anthropogenic BC INP effect despite the mentioned limitations. The identified negative forcing depends on the existence of a limited range of cirrus types, neither too depleted of homogeneously formed crystals nor too thick for thinning to cool. A substantially greater negative forcing appears unlikely, as the driving effect becomes saturated with sufficient INP presence. A sizable positive forcing may also be improbable, as this was shown to necessitate orders of magnitude more INPs than in the core model setup or an equivalently strong shift toward more heterogeneous and less homogeneous nucleation. With simulations incorporating physical and laboratory‐derived equations of ice nucleation rates over a range of plausible inputs (INPs, liquid aerosol, and updrafts), no such situation is identified as feasible. By contrast, all but one sensitivity test evidence a small to moderate negative anthropogenic BC INP forcing. The forcing could potentially negate some or all of the positive forcing caused by BC direct effects, variously estimated as 0.08–1.27 W/m^2^ (Bond et al., 2013) and 0.16–1.40 W/m^2^ (Wang et al., 2016). This could further diminish the climate mitigation potential from decreases in BC emissions (Lund et al., 2020) and may have ramifications for climate policy.

## Supporting information

Supporting Information S1

## Data Availability

Averaged output from model simulations used is available online (http://doi.org/10.5281/zenodo.3863025).
