# Supplementary material for: Global Radiative Impacts of Black Carbon Acting as Ice Nucleating Particles
Source: Geophys Res Lett. 2020 Oct 12;47(20):e2020GL089056. doi: 10.1029/2020GL089056 (PMC7757207; doi:10.1029/2020GL089056)
Supplement: Supplementary file 1 — Supporting Information S1 [file GRL-47-e2020GL089056-s001.pdf]

Supporting Information for

Global radiative impacts of black carbon acting as ice nucleating particles

Zachary McGraw[1], Trude Storelvmo[1], Bjørn Hallvard Samset[2], Camilla Weum Stjern[2]

[1] Department of Geosciences, University of Oslo, Norway

[2] Center for International Climate and Environmental Research-Oslo (CICERO), Norway

Contents of this file

Text S1
Tables S1 to S3
Figures S1 to S4

Introduction

This supplement includes tables showing runs used (Table S1) and global mean info from these (Tables S2 & S3), as well as graphics showing output from the core simulation set of this study (Figures S1-4).

Text S1 – Supplementary information on CESM simulations

Modifications were made to prevent the model from generating overly high ice crystal numbers compared to *Sourdeval et al* [2018] and strong ice cloud radiative effects relative to those of *Hong et al* [2016]. Ice crystal number density from our model control setup and from satellite retrievals are shown in Figure S3. The size threshold where ice crystals are autoconverted into snow in stratiform clouds was lowered from 400μm to 250μm, while low cloud fractions were reduced such that total cloud global shortwave, longwave, and net radiative effects roughly matched those in the EBAF Ed2.8 dataset [*Loeb et al*, 2018]. This was done by lowering the 'C8' parameter in the Cloud Layers Unified By Binormals (CLUBB) cloud macrophysics and moist turbulence parameterization from 3.0 to 1.0.

Cirrus nucleation used the *Barahona & Nenes* [2009] cirrus nucleation scheme, which replaced the default *Liu & Penner* [2005] scheme in our simulations. As in *Sullivan et al* [2016], a distribution of updrafts were fed into the scheme's nucleation processes using six-point Legendre-Gauss quadrature with updraft variability estimated from turbulent kinetic energy (TKE). However, we here used the resolved model vertical motion as the distribution mean rather than a constant value.

For clean comparisons between simulations, horizontal winds were nudged to ERA-Interim reanalysis [*Dee et al*, 2011] using a 6-hour time step. Most simulations were run for the 5-year period 2010-2014, though to avoid computational expense we only ran the sensitivity tests with cirrus radiative diagnostics for the 2-year period 2010-2011, such that the values represented in Figure 2 and Table S3 are from 2-year runs while all other values shown are for the full 5-year period.

Simulation set	Name	Description
SS1	Core set	CESM2 modified to use the *Barahona & Nenes* [2009] cirrus nucleation scheme and INP efficiencies following *Ullrich et al* [2017], with additional modifications described in Methods.
SS2	Weak updrafts	As in core, except with 0.5x the standard deviations of updrafts fed into the cirrus nucleation scheme everywhere
SS3	Strong updrafts	As in core, except with 2x the standard deviations of updrafts fed into the cirrus nucleation scheme everywhere
SS4	Low SO_4 amount	As in core, except with 0.1x the SO_4 number concentrations fed into the cirrus nucleation scheme everywhere
SS5	High SO_4 amount	As in core, except with x10 the SO_4 number concentrations fed into the cirrus nucleation scheme everywhere
SS6	All BC aged	As in core, except all BC INPs are assumed by cirrus nucleation scheme to be aged/coated, being inefficient INPs (incorporates 2x simulations with no ice nucleation on BC from SS1)
SS7	All BC fresh	As in core, except all BC INPs are assumed by cirrus nucleation scheme to be fresh/uncoated, being relatively efficient INPs (incorporates 2x simulations with no ice nucleation on BC from SS1)
SS8	High BC number	As in core, except with 10x the BC INP number concentrations fed into cirrus and mixed-phase cloud ice nucleation schemes everywhere (only in simulation with PD BC and BC INP effects on, with 3x simulations from SS1 also used here)
SS9	Low dust number	As in core, except with 0.1x the dust INP number concentrations fed into cirrus and mixed-phase cloud ice nucleation schemes everywhere
SS10	With aircraft BC	As in core, except PD simulations include aircraft BC emissions (incorporates 2x PI simulations from SS1)

Stand-alone simulation	Description
SA1	No dust or BC INPs
SA2	BC INPs x 100
SA3	BC INPs x 1000
SA4	BC INPs x 10000
SA5	BC INPs active except in stratiform cirrus
SA6	BC INPs active except in stratiform mixed-phase clouds
SA7	BC INPs active except in convective cirrus
SA8	BC INPs active except in convective mixed-phase clouds

Object	Simulation sets and stand-alone simulations used
Fig. 1	SS1
Fig. 2	SA1-4, SS1, SS8, SS9
Fig. 3	SS1-10
Fig. S1	SS1
Fig. S2	SS1
Fig. S3	SS1
Fig. S4	SS1, SS10
Table S2	SS1, SA5-8
Table S3	SS1-10

Table S1: Description of all simulation sets ('SS', upper table) and stand-alone simulations ('SA', middle) used in this study. Also listed are the simulations used to make each relevant figure and table (bottom). Each simulation set combines four simulations: PD aerosol, PI aerosol, PD aerosol without ice nucleation on BC, and PI aerosol without ice nucleation on BC. Some of these sets combined unique simulations with runs from SS1, as is noted in the descriptions. All stand-alone simulations have present-day aerosols fed into the model with input to the ice nucleation schemes varied as described, and are otherwise as in SS1.

Simulation set	Shortwave (W/m^2)	Longwave (W/m^2)	Net (W/m^2)
All BC nucleation	+0.21	-0.44	-0.23
BC stratiform cirrus nucleation	+0.12	-0.35	-0.23
BC stratiform MPC nucleation	+0.04	-0.05	-0.01
BC convective cirrus nucleation	+0.01	-0.04	-0.04
BC convective MPC nucleation	0.00	-0.02	-0.02

Table S2: BC INP radiative forcings, separated into forcings due to nucleation in each cloud type. Shown are globally averaged effective radiative forcings from all BC (anthropogenic+pre-industrial).

Simulation set name	**(i)** Base SW cirrus effect *W/m²*	**(ii)** Base LW cirrus effect *W/m²*	**(iii)** Base net cirrus effect *W/m²*	Anthropogenic BC impact on **(i)** *W/m²*	Anthropogenic BC impact on **(ii)** *W/m²*	Anthropogenic BC impact on **(iii)** *W/m²*
Control	-5.63	+9.96	+4.34	+0.07	-0.17	-0.11
Weak updrafts	-4.13	+8.16	+4.03	+0.00	-0.01	-0.01
Strong updrafts	-9.03	+14.15	+5.12	+0.32	-0.50	-0.18
Low SO_4 amount	-4.56	+9.01	+4.46	+0.06	-0.12	-0.06
High SO_4 amount	-7.18	+11.01	+3.83	+0.32	-0.36	-0.05
All BC aged	-5.80	+10.32	+4.51	-0.01	-0.05	-0.06
All BC fresh	-5.59	+9.86	+4.27	+0.04	-0.14	-0.10
High BC number	-5.48	+9.74	+4.26	+0.21	-0.40	-0.19
Low dust number	-6.53	+11.14	+4.61	+0.31	-0.51	-0.20
With aircraft BC	-5.50	+9.84	+4.35	+0.09	-0.14	-0.06

Table S3: Simulated cirrus radiative effects and associated anthropogenic BC INP impacts during the 2-year period 2010-2011. All values are global averages in W/m^2.

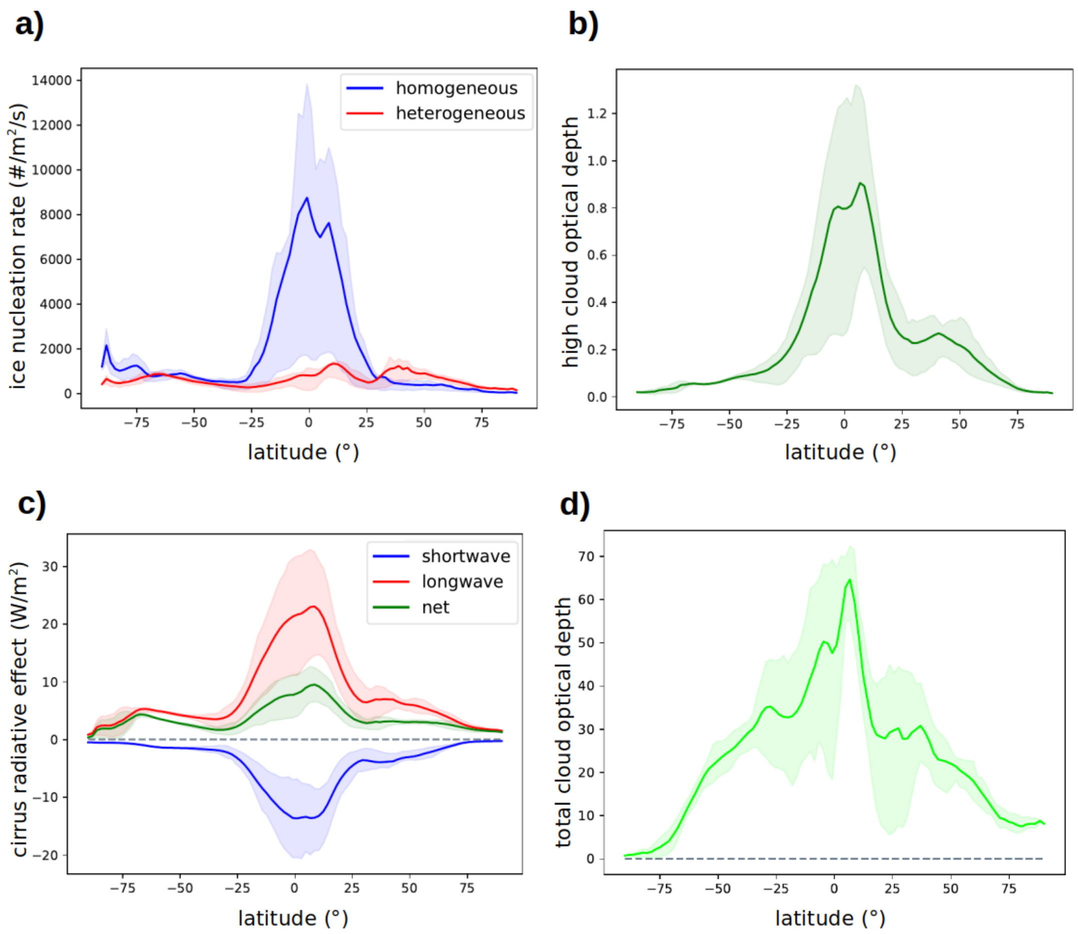

Figure S1: Simulated cloud properties, showing heterogeneous and homogeneously formed cirrus nucleation rates **(a)**, high cloud optical depth **(b)**, cirrus radiative effects **(c)**, and total cloud optical depth **(d)**. Shading shows the 25th and 75th percentiles among columns within each zonal band. BC INP impacts on the same quantities (a),(b), & (c) are shown in Figure 1 (as 1b, 1c, & 1d, respectively).

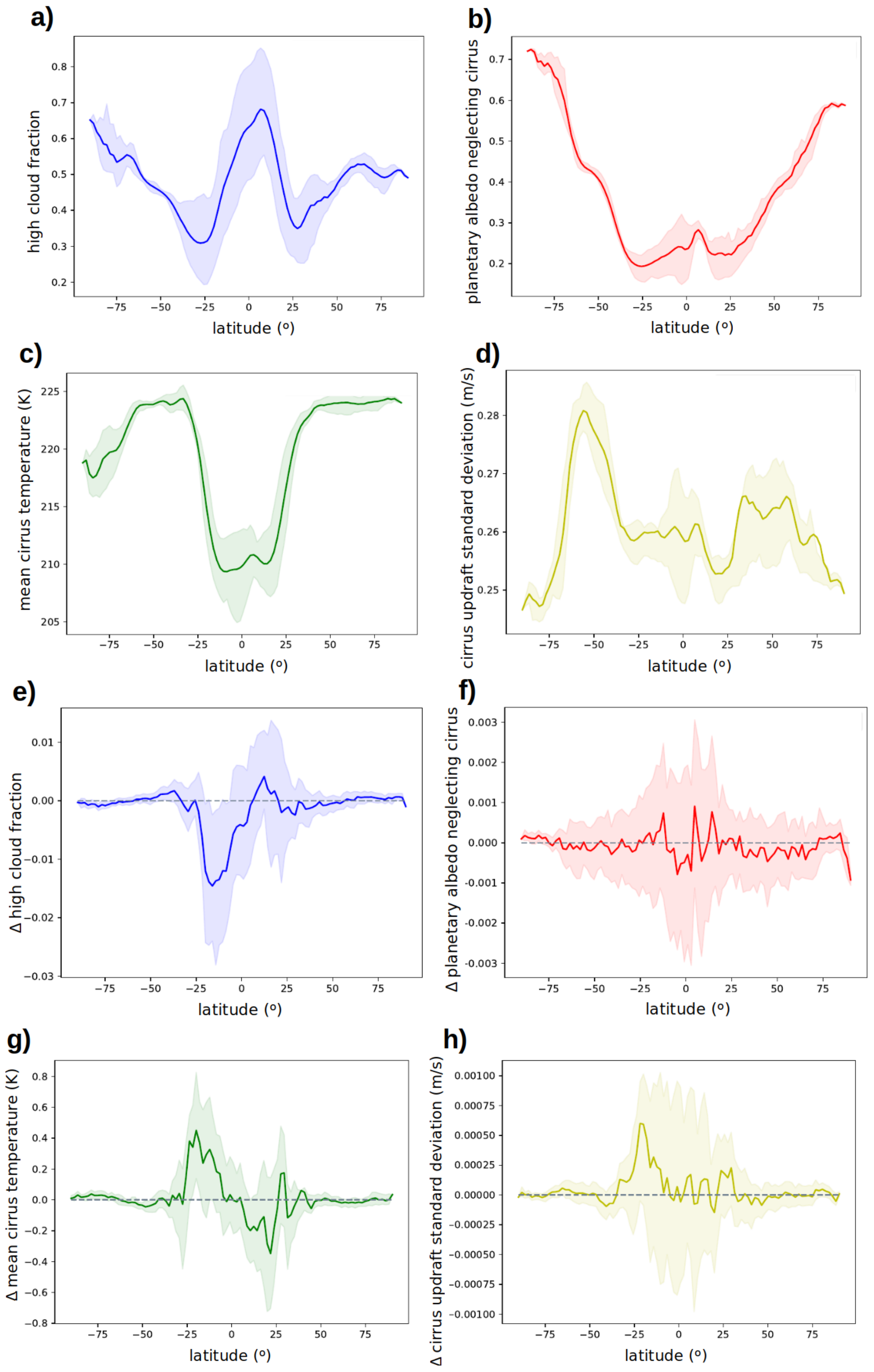

Figure S2: Cloud properties **(a,b,c,d)** and impacts of anthropogenic BC INPs **(e,f,g,h)** to these properties, showing high cloud fraction (<400mb) **(a,e)**, planetary albedo neglecting cirrus clouds **(b,f)**, mean cirrus temperature **(c,g)**, and cirrus updraft standard deviations **(d,h)**. Quantities in (c,d,g,h) were vertically averaged, weighing by cirrus cloud fraction. Shading shows the 25th and 75th percentiles among columns within each zonal band.

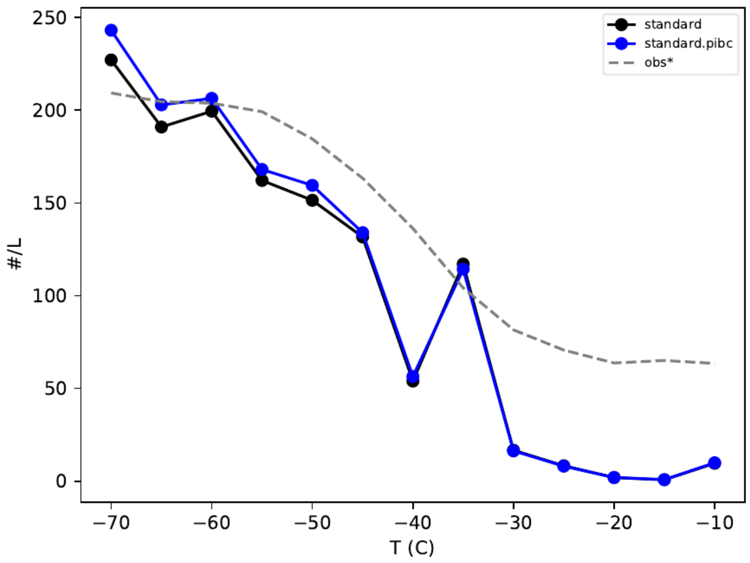

Figure S3: in-cloud number density of ice crystals >5µm, counting fully glaciated clouds on. Shown are global mean values from simulations with present day BC (black) and pre-industrial BC (blue), and the satellite retrievals of *Sourdeval et al* [2018] for comparison (grey dashed line).

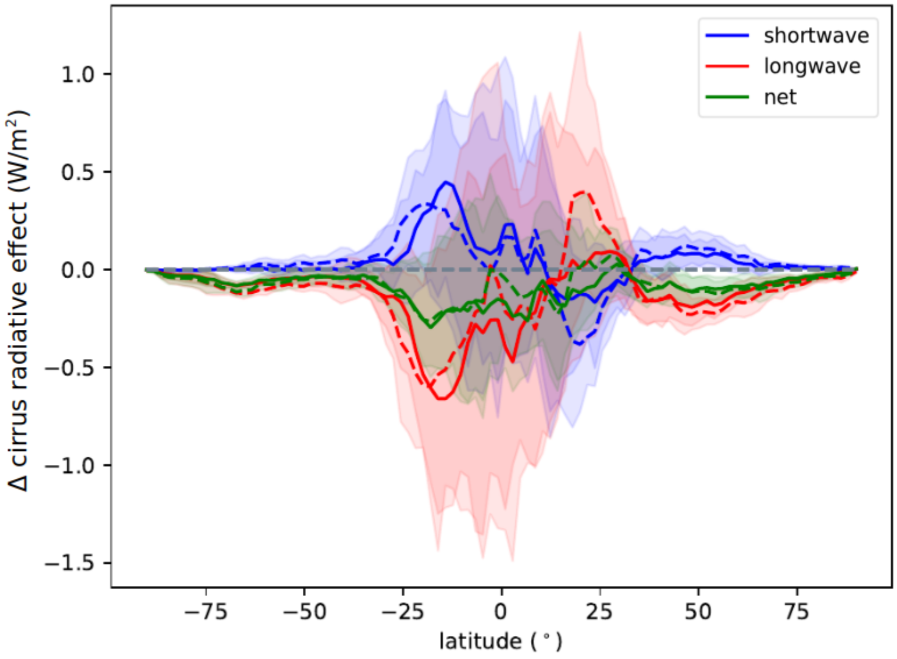

Figure S4: As in Fig. 1e but with the addition of simulations that considered changes to aircraft soot emissions (dotted lines) in addition to sources represented throughout this study.

References from the Supporting Information

Barahona, D., & Nenes, A. (2009). Parameterizing the competition between homogeneous and heterogeneous freezing in ice cloud formation-polydisperse ice nuclei. *Atmos. Chem. Phys.* 9, 16. https://doi.org/10.5194/acp-9-5933-2009

Dee, D. P., Uppala, S. M., Simmons, A. J., Berrisford, P., Poli, P., Kobayashi, S., et al. (2011). The ERA-Interim reanalysis: Configuration and Q12 performance of the data assimilation system. *Quarterly Journal of the Royal Meteorological Society*, 137(656), 553–597. https://doi.org/10.1002/qj.828

Hong, Y., Liu, G., & Li, J. L. (2016). Assessing the radiative effects of global ice clouds based on CloudSat and CALIPSO measurements. *J. Clim.* 29, 7651-7674 . https://doi.org/10.1175/JCLI-D-15-0799.1

Liu, X., & Penner, J. E. (2005). Ice nucleation parameterization for global models. *Meteorologische Zeitschrift*, 14(4), 499-514.

Loeb, N. G., Doelling, D. R., Wang, H., Su, W., Nguyen, C., Corbett, J. G., …et al. (2018). Clouds and the earth’s radiant energy system (CERES) energy balanced and filled (EBAF) top-of-atmosphere (TOA) edition-4.0 data product. *Journal of Climate*, 31(2), 895-918. https://doi.org/10.1175/JCLI-D-17-0208.1

Sourdeval O, Gryspeerdt E, Krämer M, Goren T, Delanoë J, Afchine A, Hemmer F, Quaas J. (2018). Ice crystal number concentration estimates from lidar–radar satellite remote sensing–Part 1: Method and evaluation. *Atmospheric Chemistry and Physics*. 2018;18(19):14327-50. https://dx.doi.org/10.5194/acp-18-14327-2018

Sullivan, S. C., Morales Betancourt, R., Barahona, D., & Nenes, A. (2016). Understanding cirrus ice crystal number variability for different heterogeneous ice nucleation spectra. *Atmospheric Chemistry and Physics*, 16, 2611-2629. https://doi.org/10.5194/acp-16-2611-2016
